# Estudio Multicéntrico de la Frecuencia de Oclusión de Gran Vaso (OGV) en Pacientes con Ictus Minor

**DOI:** 10.31083/RN33477

**Published:** 2025-04-03

**Authors:** Pablo Ros-Arlanzón, Diego Corona García, Raquel Hernández Lorido, Isabel Beltrán Blasco, José Tembl Ferrairo, Cristina Soriano Soriano, Nicolás López Hernández

**Affiliations:** ^1^Departamento de Neurología, Hospital General Universitario Dr. Balmis, 03010 Alicante, España; ^2^Instituto de Investigación Sanitaria y Biomédica de Alicante (ISABIAL), 03010 Alicante, España; ^3^Departamento de Neurología, Hospital Universitario y Politécnico La Fe, 46026 Valencia, España; ^4^Departamento de Neurología, Hospital General de Castellón, 12004 Castellón, España

**Keywords:** ictus, ictus minor, angiografía por tomografía computarizada, oclusión de gran vaso, stroke, minor stroke, computed tomography angiography, large vessel occlusion

## Abstract

**Introducción:**

El manejo del ictus isquémico minor presenta desafíos significativos, debido a la variabilidad en la aplicación de protocolos de neuroimagen y tratamiento endovascular (TEV). La ausencia de consenso sobre la indicación de la angiografía por tomografía computarizada (angioTC) en estos casos subraya la importancia de investigar la prevalencia y las implicaciones clínicas de la oclusión de gran vaso en esta población.

**Metodología:**

Análisis del registro multicéntrico CODICT en pacientes con ictus isquémico minor (National Institutes of Health Stroke Scale (NIHSS) ≤5) atendidos en centros terciarios de atención al código ictus (CI) de la Comunidad Valenciana en el periodo 01/07/2020–30/11/2023. Se evaluó la frecuencia de oclusión de gran vaso (OGV), definida como oclusiones en carótida interna, arteria vertebral, basilar, y segmentos críticos de la arteria cerebral media (M1, M2), anterior (A1, A2) y posterior (P1, P2), mediante angioTC.

**Resultados:**

Se identificaron un total de 5473 activaciones de CI en el periodo de estudio. Un total de 833 pacientes sufrieron un ictus isquémico minor. El 17,5% (n = 146) de los ictus minor mostraron una OGV en el angioTC. El 48,6% (n = 71) de los pacientes con ictus minor y OGV fueron sometidos a TEV. Los vasos más frecuentemente ocluidos fueron la arteria cerebral media (ACM) en sus segmentos M1 y M2 ambos en el 35,6% (n = 52) de los casos. Sin embargo, el vaso más frecuentemente tratado mediante TEV fue M1 en el 29,5% (n = 43), seguido por M2 en el 10,9% (n = 16) de los casos.

**Conclusiones:**

Este estudio pone de manifiesto la importancia de la realización de angioTC en todo paciente que cumpla criterios de activación de Código Ictus, independientemente de la gravedad clínica. La presencia de OGV cambió el manejo clínico en casi la mitad de los pacientes con ictus minor y OGV.

## Abstract (en)

**Background:**

The management of minor ischemic stroke presents significant challenges due to variability in the application of neuroimaging protocols and endovascular treatment (EVT). The lack of consensus on the need for computed tomography angiography (CTA) in these cases highlights the importance of investigating the prevalence and clinical implications of large vessel occlusion (LVO) in this population.

**Methods:**

Analysis of the multicenter CODICT registry in patients with minor ischemic stroke (National Institutes of Health Stroke Scale (NIHSS) score ≤5) treated at tertiary stroke alert (SA) centers in the Valencian Community between July 1, 2020 and November 30, 2023. The frequency of LVO, defined as occlusions in the internal carotid artery, vertebral artery, basilar artery, and critical segments of the middle (M1, M2), anterior (A1, A2), and posterior (P1, P2) cerebral arteries, was evaluated using CTA

**Results:**

A total of 5473 SA activations were identified during the study period. A total of 833 patients suffered a minor ischemic stroke. LVO was observed in 17.5% (n = 146) of minor strokes on CTA. EVT was performed in 48.6% (n = 71) of patients with minor stroke and LVO. The most frequently occluded vessels were the middle cerebral artery (MCA) in its M1 and M2 segments, both in 35.6% (n = 52) of cases. However, the vessels most frequently treated with EVT were in the M1, in 29.5% (n = 43) of cases, followed by the M2, in 10.9% (n = 16) of cases.

**Conclusions:**

This study highlights the importance of performing CTA in all patients who meet SA activation criteria, regardless of clinical severity. The presence of LVO changed the clinical management in almost half of the patients with minor stroke and LVO.

## 1. Introducción

El ictus isquémico representa una de las principales causas de discapacidad y mortalidad a nivel mundial. Dada su potencial gravedad en términos de morbimortalidad, definir un ictus como “minor” puede ser controvertido. El término ictus minor se emplea habitualmente para pacientes con ictus con síntomas leves y no incapacitantes [1]. Sin embargo, el ictus minor, a pesar de su denominación puede evolucionar rápidamente a un escenario más grave o dejar secuelas a priori “leves” que supongan un impacto importante en la calidad de vida de los pacientes [2].

La literatura médica actual revela una falta de consenso respecto al tratamiento óptimo de los pacientes con ictus minor, especialmente en el contexto de una oclusión de gran vaso (OGV) [3, 4, 5]. La identificación de estos casos es crucial, dado que el tratamiento endovascular (TEV) puede ser potencialmente beneficioso en pacientes con OGV, incluso cuando los síntomas son inicialmente leves.

Algunos estudios sugieren que la administración temprana de tratamientos recanalizadores, como el TEV, podría mejorar significativamente los resultados en estos pacientes, mientras que otros advierten sobre los riesgos de intervenciones innecesarias o prematuras [3]. Sin embargo, la realización rutinaria de angiografía por tomografía computarizada (angioTC) en estos pacientes es todavía un tema de debate. Las principales guías muestran una recomendación fuerte para la realización de angioTC en pacientes con ictus isquémico con afectación clínica moderada o grave, pero también se recomienda realizar en cualquier paciente con ictus isquémico en la fase aguda, lo que incluiría a pacientes con ictus minor [6].

En este estudio, se analiza la presencia de OGV en pacientes con ictus isquémico y síntomas leves en 3 centros terciarios de la Comunidad Valenciana, con el objetivo de conocer su frecuencia.

## 2. Metodología

Se realizó un análisis retrospectivo del registro multicéntrico CODICT. Se incluyeron pacientes atendidos en tres centros terciarios de la Comunidad Valenciana (HGUDB, HGUC y HUPLF) con criterios de activación de código ictus (CI) y diagnóstico de ictus isquémico minor, definido como una puntuación del *National Institutes of Health Stroke Scale* (NIHSS) menor o igual a 5. La definición de ictus isquémico y las diferentes etiologías se realizaron en base a las recomendaciones de la Sociedad Española de Neurología [7]. Todos los pacientes, independientemente de la gravedad clínica, fueron sometidos a una angioTC como parte de su evaluación inicial. El periodo de estudio abarcó del 01/07/2020 al 30/11/2023.

### 2.1 Criterios de Exclusión

Se excluyeron los casos en los que se desactivó el CI, en los que la evolución de los síntomas fue superior a 24 horas, con una puntuación en la escala de discapacidad de escala de Rankin modificada (mRS) mayor a 2, con diagnóstico final de ictus hemorrágico, o con presencia de cuadros clínicos imitadores de ictus (conocidas como *stroke mimic*).

### 2.2 Definición de Oclusión de Gran Vaso

Se definió la OGV como la oclusión de las siguientes arterias cerebrales: carótida interna, arteriva vertebral, arteria basilar, arteria cerebral media (ACM) en segmentos M1 y M2, arteria cerebral anterior (ACA) en segmentos A1 y A2 y arteria cerebral posterior (ACP) en segmentos P1 y P2.

### 2.3 Recogida de Variables y Consideraciones Éticas

Como se describió en un estudio anterior, las variables fueron registradas de manera prospectiva utilizando la aplicación CODICT por el neurólogo responsable en el momento de activar el CI [8]. El registro incluyó tiempos desde la activación del CI, variables clínicas desde el ingreso y durante la estancia en la Unidad de Ictus (UIC), y la situación funcional a los 3 meses en los pacientes sometidos a TEV. Los datos se exportaron posteriormente en una hoja de cálculo de Excel para su análisis. Se cumplieron con los estándares éticos y de buena práctica clínica, el registro CODICT se trata de un registro anonimizado que cuenta con la aprobación ética del Comité de Ética de la Investigación con Medicamentos del Departamento de Salud de Alicante-Hospital General (Acta 2020-06).

### 2.4 Análisis Estadístico

**Fig. 1.**
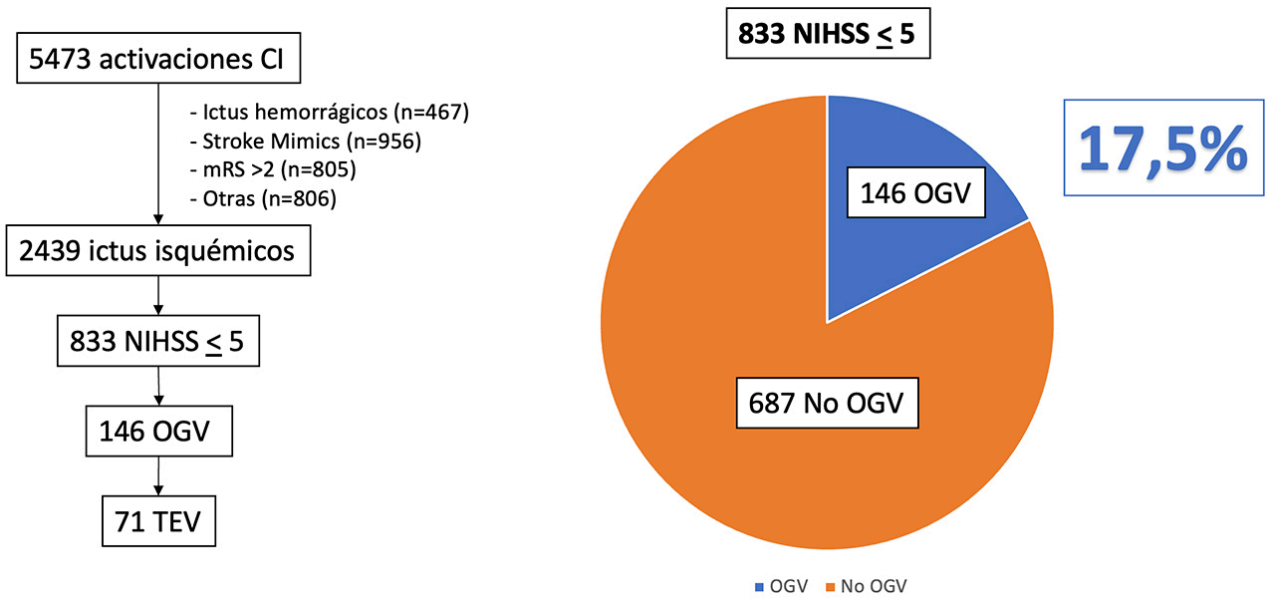
**Flujo de pacientes y proporción de pacientes con ictus minor con oclusión de gran vaso**. CI, código ictus; OGV, oclusión de gran vaso; TEV, tratamiento endovascular; mRS, escala de Rankin modificada; NIHSS, National Institutes of Health Stroke Scale.

El análisis estadístico se llevó a cabo utilizando el software R (versión 4.2.1, R Foundation for Statistical Computing, Vienna, Austria). Las variables cualitativas se describieron mediante frecuencias y porcentajes relativos al subgrupo directamente superior, tal como se establecen en la Fig. 1. Las variables cuantitativas se expresaron en forma de media y desviación estándar (DE). Para todas las pruebas estadísticas de contraste de hipótesis se aceptó un valor de significación de 0,05.

## 3. Resultados

### 3.1 Frecuencia de Ictus Isquémico Minor y de Oclusión de Gran Vaso en Pacientes con Ictus Minor

Durante el período de estudio, se registraron un total de 5473 activaciones de CI en los centros participantes. De estas activaciones, 2439 (44,56%) cumplieron verdaderamente criterios de activación de CI y de ellos, 833 (34,2%) casos correspondieron a pacientes que sufrieron un ictus isquémico minor.

De los 833 pacientes con ictus minor, se identificaron 146 con una OGV en el estudio de angioTC, lo que supuso una proporción del 17,5% de los pacientes con ictus minor. Entre estos pacientes con OGV, un 71 (48,6%) fueron sometidos a TEV (ver Fig. 1). Las características clínicas y demográficas de los pacientes pueden verse en la Tabla 1.

**Tabla 1.** **Características clínicas de los pacientes con ictus isquémico minor**.

		Ictus minor	No OGV	Si OGV	*p*
n		833	687	146	
Unidad (%)					0,234
	- Alicante	426 (51,1)	342 (49,8)	84 (57,5)	
	- Castellón	100 (12,0)	85 (12,4)	15 (10,3)	
	- La Fe	307 (36,9)	260 (37,7)	47 (32,9)	
Edad (media (DE))		67,38 (12,96)	67,64 (12,88)	66,16 (13,30)	0,211
Sexo = hombre (%)		538 (64,6)	455 (66,2)	83 (56,8)	0,040
Alcoholismo = si (%)		40 (4,8)	36 (5,2)	4 (2,7)	0,285
Tabaquismo = si (%)		219 (26,3)	173 (25,2)	46 (31,5)	0,141
HTA = si (%)		539 (64,7)	464 (67,5)	75 (51,4)	<0,001
Diabetes = si (%)		253 (30,4)	219 (31,9)	34 (23,3)	0,051
Dislipemia = si (%)		449 (53,9)	377 (54,9)	72 (49,3)	0,257
FA = si (%)		94 (11,3)	73 (10,6)	21 (14,4)	0,246
Ictus previos = si (%)		130 (15,6)	115 (16,7)	15 (10,3)	0,067
mRS previo					0,015
	∙ 0	645 (77,4)	521 (75,8)	124 (84,9)	
	∙ 1	139 (16,7)	119 (17,3)	20 (13,7)	
	∙ 2	49 (5,9)	47 (6,8)	2 (1,4)	
FIV (%)		140 (16,8)	89 (13,0)	51 (34,9)	<0,001
TEV (%)		71 (8,5)	0 (0,0)	71 (48,6)	<0,001
NIHSS basal (mediana [RIC])		3 (1–4)	2 (1–4)	4 (2–5)	<0,001
Complicaciones neurológicas		30 (3,6)	20 (2,9)	10 (6,8)	0,038
Complicaciones sistémicas		79 (9,5)	62 (9,0)	17 (11,6)	0,409
Exitus letalis durante el ingreso		14 (1,7)	10 (1,5)	4 (2,7)	0,458
Etiología					<0,001
	∙ Aterotrombótico	165 (23,0)	117 (20,2)	48 (34,5)	
	∙ Cardioembolico	132 (18,4)	88 (15,2)	44 (31,7)	
	∙ Lacunar	155 (21,6)	152 (26,3)	0 (0)	
	∙ Otras	381 (45,7)	331 (47,9)	54 (36,9)	

DE, desviación estándar; FA, fibrilación auricular; FIV, fibrinólisis intravenosa; HTA, hipertensión arterial; RIC, rango intercuartílico. En (%) se indica el porcentaje en proporción a la categoría de cada columna.

En cuanto al tratamiento trombolítico con fibrinolisis intravenosa (FIV) se observó una mayor tasa de FIV en los pacientes con ictus minor y OGV frente a los pacientes con ictus minor y sin OGV (34,9% vs. 13% *p*-valor < 0,001).

### 3.2 Análisis de Los Pacientes con Ictus Minor y Oclusión de Gran Vaso

En la Tabla 2 pueden encontrarse las características clínicas de los pacientes con ictus minor y OGV así como la topografía de las OGV encontradas.

**Tabla 2.** **Características clínicas y topografía de oclusión de gran vaso en los pacientes con ictus isquémico minor y oclusión de gran vaso**.

		OGV	TEV (-)	TEV (+)	*p*
n		146	75	71	
Unidad (%)					0,317
	- Alicante	84 (57,5)	46 (61,3)	38 (53,5)	
	- Castellón	15 (10,3)	9 (12,0)	6 (8,5)	
	- La Fe	47 (32,2)	20 (26,7)	27 (38,0)	
Edad (media (DE))		66,16 (13,30)	65,55 (14,15)	66,82 (12,40)	0,566
Sexo = hombre (%)		83 (56,8)	45 (60,0)	38 (53,5)	0,533
Alcoholismo = si (%)		4 (2,7)	3 (4,0)	1 (1,4)	0,652
Tabaquismo = si (%)		46 (31,5)	22 (29,3)	24 (33,8)	0,687
HTA = si (%)		75 (51,4)	39 (52,0)	36 (50,7)	1,000
Diabetes = si (%)		34 (23,3)	20 (26,7)	14 (19,7)	0,425
Dislipemia = si (%)		72 (49,3)	34 (45,3)	38 (53,5)	0,410
FA = si (%)		21 (14,4)	8 (10,7)	13 (18,3)	0,280
Ictus previos = si (%)		15 (10,3)	10 (13,3)	5 (7,0)	0,328
mRS previo					0,422
	∙ 0	124 (84,9)	61 (81,3)	63 (88,7)	
	∙ 1	20 (13,7)	13 (17,3)	7 (9,9)	
	∙ 2	2 (1,4)	1 (1,3)	1 (1,4)	
FIV (%)		51 (34,9)	27 (36,0)	24 (33,8)	0,917
NIHSS basal (mediana [RIC])		4 [2–5]	3 [2–5]	4 [3–5]	0,201
Complicaciones neurológicas		10 (6,8)	4 (5,3)	6 (8,5)	0,676
Complicaciones sistémicas		17 (11,6)	8 (10,7)	9 (12,7)	0,904
Exitus letalis durante el ingreso		4 (2,7)	1 (1,3)	3 (4,2)	0,574
Etiología					0,686
	∙ Aterotrombótico	48 (34,5)	24 (34,8)	24 (34,3)	
	∙ Cardioembolico	44 (31,7)	19 (27,5)	25 (35,7)	
	∙ Otras	54 (36,9)	32 (42,7)	22 (30,9)	
Topografía OGV*					
	∙ Basilar	9	7	2	
	∙ AV	0	5	3	
	∙ ACI cervical	30	19	11	
	∙ ACI intracranial	7	1	6	
	∙ T/L carotídea	4	0	4	
	∙ M1	52	9	43	
	∙ M2	52	36	16	
	∙ P1	4	4	0	
	∙ P2	5	4	1	

ACI, arteria carótida interna; AV, arteria vertebral. En (%) se indica el porcentaje en proporción a la categoría de cada columna. *Se encontraron múltiples oclusiones en >1 vaso y oclusiones en tándem intracraneal-extracraneal, pero los resultados se expresan por afectación de cada vaso individual. Dado el solapamiento, la suma de las OGV es mayor que el número de pacientes. TEV (-) y (+): se refieren, respectivamente, al subgrupo de pacientes que no recibieron tratamiento endovascular y al subgrupo que sí lo recibió.

El análisis de los vasos ocluidos reveló que los segmentos M1 y M2 de la ACM fueron los sitios de oclusión más frecuentes, registrados ambos en 52 (35,6%) de los casos con ictus minor y OGV. En cuanto al TEV, el segmento M1 de la ACM resultó ser el vaso más frecuentemente tratado, con 43 (29,5%) pacientes con ictus minor y OGV sometidos a TEV. En contraste, el segmento M2 de la ACM, fue tratado con TEV en una proporción significativamente menor, con solo 16 (10,9%) pacientes.

## 4. Discusión

Clásicamente, los ictus isquémicos lacunares son el subtipo etiológico que presenta un mejor pronóstico funcional a corto plazo y que más frecuentemente se presenta como un ictus minor, así mismo, se ha señalado que una proporción de estos pacientes tienen otras etiologías [9]. Nuestros hallazgos indican una prevalencia considerable de OGV entre los pacientes con ictus minor. Los pacientes con ictus minor suponen una proporción significativa de los casos de ictus isquémico agudo que se atienden en los servicios de urgencias [10]. A pesar de su aparente menor gravedad, el ictus minor puede conllevar retrasos en la administración del tratamiento trombolítico [11], así como peores resultados en términos de discapacidad cuando se comparan con ictus isquémicos agudos de mayor gravedad clínica [10, 12].

Una posible explicación para los peores resultados en los pacientes con ictus minor es la existencia, todavía hoy, de limitaciones en el acceso a estudios de neuroimagen multimodal. La percepción errónea de estos ictus como afecciones de menor relevancia clínica podría influir en la decisión de los médicos o de los servicios de urgencias y radiología de no realizar dichos estudios, lo que impacta negativamente en el diagnóstico y tratamiento oportuno.

Sin embargo, nuestros resultados evidencian que la frecuencia de OGV en estos pacientes es notable, presentándose en casi 1 de cada 5 pacientes con ictus isquémico minor con criterios de activación de código ictus. La literatura reporta que la frecuencia de OGV en este contexto varía ampliamente, desde un 4% hasta un 24,5%, dependiendo de la cohorte estudiada [12, 13, 14, 15]. La frecuencia de OGV en nuestra serie y en otras similares puede estar parcialmente incrementada respecto a otras series, puesto que metodológicamente quedan excluidos los casos de *stroke-mimic*.

Aunque no existen ensayos clínicos específicos que evalúen directamente los resultados del TEV en pacientes con OGV e ictus minor, los estudios observacionales y subanálisis de ensayos clínicos originales sugieren un beneficio del TEV al menos en los casos de oclusión en M1 [3, 4, 5]. En nuestro estudio, los pacientes con ictus minor y OGV sometidos a TEV no presentaron una mayor incidencia de complicaciones en comparación con aquellos que no fueron tratados con esta intervención. Es importante destacar que los pacientes que recibieron TEV (Tabla 2) tenían un perfil clínico más grave (NIHSS basal 2 [1–4] vs. 4 [2–5]; *p* < 0,001), lo que puede sugerir la seguridad del procedimiento en este subgrupo de pacientes.

Una limitación importante de nuestro estudio es la ausencia de datos de seguimiento a largo plazo en los pacientes que no recibieron TEV lo que impide una evaluación completa de la seguridad y la efectividad del TEV en esta población.

Independientemente de si se realiza TEV o no en pacientes con ictus minor y OGV, estos pacientes requieren una monitorización estrecha debido al riesgo de empeoramiento de los síntomas o progresión del ictus, lo que constituiría una indicación clara para el TEV urgente [16, 17]. En este sentido, resulta fundamental mejorar el acceso a estudios de neuroimagen multimodal para facilitar un diagnóstico preciso y oportuno en este grupo de pacientes. Asimismo, se hace evidente la necesidad de realizar ensayos clínicos que evalúen específicamente la efectividad del TEV en pacientes con ictus isquémico minor y OGV [18].

Este estudio resalta la prevalencia significativa de OGV en pacientes con ictus isquémico minor y sugiere la posible seguridad del TEV en estos casos. No obstante, la falta de evidencia robusta sobre los beneficios a largo plazo, junto con las barreras en el acceso a tecnologías de imagen avanzadas, subrayan la necesidad urgente de revisar las prácticas actuales y promover investigaciones adicionales en este campo. Líneas futuras de investigación deben centrarse en estudios prospectivos que evalúen la seguridad y la efectividad de las terapias de recanalización en pacientes con ictus minor y OGV.

## 5. Conclusión

Este estudio pone de manifiesto la importancia de la realización de angioTC en todo paciente que cumpla criterios de activación de Código Ictus, independientemente de la gravedad clínica o la clasificación como ictus minor. La presencia de OGV en pacientes con ictus minor, supuso un cambio en la toma de decisiones clínicas en casi la mitad de este subgrupo de pacientes. Aunque la presencia de oclusión de gran vaso puede influir en las decisiones clínicas, actualmente no existe un consenso claro sobre el mejor tratamiento para los pacientes con ictus minor y oclusión de gran vaso. Esto subraya la necesidad de futuras investigaciones para definir las estrategias de manejo óptimas en esta subpoblación.

## Data Availability

Los conjuntos de datos utilizados y analizados durante el presente estudio están disponibles a través del autor de correspondencia previa solicitud razonable.

## Abreviaturas

angioTC, angiografía por tomografía computarizada angio; OGV, oclusión de gran vaso; TEV, tratamiento endovascular.

## Contribuciones de los Autores

PRA ideó el proyecto, recolectó y analizó los datos, preparó la versión inicial del manuscrito y las subsiguientes correcciones. DCG recolectó datos y participó en el análisis e interpretación de los resultados, así como en revisiones críticas del manuscrito. RHL, IBB, JTF y CSS coordinaron la recolección y validación de los datos y la revisión crítica del manuscrito. NLH dirigió el proyecto, recolectó y depuró los datos, coordinó el proyecto y participó en la revisión crítica del artículo. Todos los autores leyeron y aprobaron el manuscrito final. Todos los autores han contribuido significativamente al trabajo y han acordado ser responsables de todos los aspectos del mismo.

## Aprobación Ética y Consentimiento Informado

El registro CODICT fue aprobado por el Comité de Ética de la Investigación con Medicamentos del Departamento de Salud de Alicante - Hospital General, durante su sesión del 27 de mayo de 2020 (Acta 2020-06). Dado que se trata de un subanálisis de un registro con datos completamente anonimizados (registro CODICT), no se requirió un consentimiento informado adicional para participar en este estudio. Este estudio se llevó a cabo de acuerdo con las directrices de la Declaración de Helsinki.

## Agradecimientos

El grupo CODICT participó activamente en la recolección de los datos. Al grupo de estudio del registro CODICT: Carlos Aledo Sala, Luis Moreno Navarro, Mònica Farrerons Llopart, Manuel Dieter Warnken Miralles, Pau Mahiques Ochoa, Carlos Lapeña López, Elisa Ginés Murcia, Lourdes Ruiz-Escribano Menchén, Barbara Povedano Margarit, Pedro Barredo Benito, Ana Fríes Ramos, Silvia Martí Martínez, Rosa María Sánchez Pérez, Carmen Díaz Marín, Gerardo Fortea Cabo, Luis Morales Caba, Irene Escudero Martínez, Diana Tarruella Hernandez, Isabel Vielba, Lucas Barea Moya, Jesús Jiménez Jiménez, Luis Monrós Giménez, Manuel Pedrero Prieto, Elena Navarro Mocholi, Carlos Benlloch López, Jose Román Alcañiz, Jorge Ferrando Bareberá, Aaron Martínez Gimeno, Héctor Aparicio Collado, Javier Arnau Barrés, María Bas Abad, Helena Benetó Andrés, Sara Blanco Madera, Antonio Belenguer Benavides, Berta Claramonte Clausell, Marisol Campillo Alpera, Mónica Fortanet García, Marina Martínez Molina, Ana Monclús Beclua, Manuel Peinazo Arias, Julia Renau Lagranja, Natalia Ruiz Nieto, Lidia Salais López, Carlos Vilar Fabra, Rosa Vilar Ventura, Ana del Villar Igea, Pilar Yanguas Ramírez, Anabel Zahonero Férriz.

## Financiación

Esta investigación no recibió financiación externa.
